# Managing exercise food induced anaphylaxis

**DOI:** 10.1186/2045-7022-1-S1-S49

**Published:** 2011-08-12

**Authors:** Esben Eller

**Affiliations:** 1 Odense University Hospital, Department of Dermatology and Allergy Center, Odense, Denmark

## 

Food dependent exercise induced anaphylaxis (FDEIA) is an IgE mediated adverse reaction to specific foods, where symptoms only appear in combination with physical activity. In absence of exercise, subjects with FDEIA tolerate the offending food. FDEIA can, as the nomenclature indicates, be life threatening, but most common symptoms are urticaria, flushing, pruritus and angioedema. The mechanism behind FDEIA is unclear, but a number of hypotheses have been proposed, such as altered GI permeability, blood distribution or activation of mucosal mast cells. Managing FDEIA is characterized by avoidance of the offending food in combination whit physical activity. However, in order to give proper guidelines for managing FDEIA, a precise diagnosis is crucial. The diagnosis of FDEIA is based on case history, relevant sensitization, exclusion of cholinergic urticaria and “regular” non-exercise induced food allergy and finally reproduced by an exercise test. Different foods are known to elicit FDEIA, wheat being the most common. The aetiology of wheat dependent exercise induced anaphylaxis (WDEIA) is better understood than symptoms caused by other allergens. The wheat protein component Ω-5 Gliadin is known to be involved in WDIEA, but the precise role, clinical relation and relevance of in-vivo/vitro measurement is unclear. Further, the type and intensity of the exercise as well as the kinetics and time perspective need further focus. Food processing such as heat treatment, cooking and baking also influences the diagnosis and thereby the management of exercise induced food anaphylaxis. Standard diagnostic procedure in order setting include initial careful case history followed by skin prick testing with various wheat extracts, measurement of specific IgE to wheat and available wheat components followed by a histamine release from basophils, stimulated with a variety of raw and processed wheat preparations. Ultimately, a standardised treadmill exercise is performed.

